# A Note on Lobelia

**Published:** 1888-05

**Authors:** C. Carleton Smith


A NOTE ON LOBELIA.—Extreme *tenderness* over the sacrum. She cannot bear even the pressure of a soft pillow; she cries out if any attempt is made to touch the part. She sits up in bed leaning forward to avoid contact with the bedclothes. After each vomiting spell, she breaks out all over with *sweat*, followed by a sensation as if *thousands of needles* were piercing her skin, from *within outward*. C. CARLETON SMITH.

